# Comparison of attenuated and virulent West Nile virus strains in human monocyte-derived dendritic cells as a model of initial human infection

**DOI:** 10.1186/s12985-015-0279-3

**Published:** 2015-03-22

**Authors:** Daniel J Rawle, Yin Xiang Setoh, Judith H Edmonds, Alexander A Khromykh

**Affiliations:** Australian Infectious Diseases Research Centre, School of Chemistry and Molecular Biosciences, The University of Queensland, St Lucia, Queensland, 4072 QLD Australia

**Keywords:** West Nile virus, Dendritic cells, Monocyte, Pathogenesis, Virulence

## Abstract

**Background:**

The human-pathogenic North American West Nile virus strain (WNV_NY99_), responsible for the outbreak in New York city in 1999, has caused 41000 infections and 1739 human deaths to date. A new strain of West Nile virus emerged in New South Wales, Australia in 2011 (WNV_NSW2011_), causing a major encephalitic outbreak in horses with close to 1000 cases and 10-15% mortality. Unexpectedly, no human cases have so far been documented.

**Findings:**

We report here, using human monocyte-derived dendritic cells (MoDCs) as a model of initial WNV infection, that the pathogenic New York 99 WNV strain (WNV_NY99_) replicated better than WNV_NSW2011_, indicative of increased viral dissemination and pathogenesis in a natural infection. This was attributed to suppressed viral replication and type I interferon (IFN) response in the early phase of WNV_NY99_ infection, leading to enhanced viral replication at the later phase of infection. In addition, WNV_NY99_ induced significantly more pro-inflammatory cytokines in MoDCs compared to WNV_NSW2011_.

**Conclusions:**

Our results suggest that the observed differences in replication and induction of IFN response between WNV_NY99_ and WNV_NSW2011_ in MoDCs may be indicative of their difference in virulence for humans.

## Findings

West Nile virus (WNV) is the leading cause of arboviral encephalitis in the Americas with over 41000 infections and 1739 human deaths (http://www.cdc.gov/westnile/index.html, accessed 25/11/2014) since the outbreak of a human virulent strain in New York in 1999 (WNV_NY99_ strain) [1,2]. There is currently no effective treatment or approved WNV vaccine for use in humans. A strain of WNV called Kunjin (WNV_KUN_) has been circulating in Australia since it was discovered in 1960, and have caused very few symptomatic infections in humans or horses [3]. This was until a new strain of WNV emerged in New South Wales in 2011 (WNV_NSW2011_) causing a major encephalitic outbreak in over 1000 horses [4,5]. WNV_NSW2011_ has likely emerged through mutations of previously circulating WNV_KUN_, and gained at least two known virulence determinants found in the human-pathogenic WNV_NY99_ strain [4]; i) the N-linked glycosylation on the E protein associated with increased virulence in mice [6], and ii) the phenylalanine residue at position 653 of NS5 which is a potent inhibitor of STAT1 phosphorylation [7].

The horses affected by the WNV_NSW2011_ outbreak presented with similar clinical symptoms to horses infected with WNV_NY99_, therefore it was rather unexpected that the WNV_NSW2011_ outbreak had no reported human cases. This prompted us to investigate the viral growth kinetics and immune induction profiles of WNV_NSW2011_ compared to WNV_NY99_ using cultures of primary human dendritic cells (DCs) as a model of initial infection in humans [8-11]. Human MoDCs were used as an *ex vivo* model of initial WNV infection in this study, because it has been shown that large numbers of bone marrow monocytes differentiate into DCs soon after WNV infection in the dermis [12]. Human MoDCs have been previously used to show that WNV replication was required for type I IFN induction, and this was a result of IRF3 translocation to the nucleus after dsRNA stimulation of RIG-1, MDA5 or TLR3 [13]. While comparison of WNV strains has been previously performed in DCs to show that WNV strains with glycosylated envelope (E) protein have increased infection and replication rates [14], WNV_NY99_ and WNV_NSW2011_ both have glycosylated E [4], and are therefore expected to be equally efficient in viral entry into MoDCs through attachment to DC-SIGN or DC-SIGNR receptors [11]. Therefore, additional differences must exist that result in either productive infection of DCs or effective viral clearance by the innate immune response.

To investigate this, we used MoDCs by isolating peripheral blood mononuclear cells (PBMCs) from human buffy coat from three donors, which were tested to be negative for arboviral infections, then performed CD14+ magnetic selection to isolate monocytes. The isolated monocytes were cultured with GM-CSF and IL-4 for 6 days to differentiate into immature MoDCs, which were then matured upon WNV infection represented by increased CD80 and CD86 expression. Briefly, at 48 hours post infection (hpi), 78.7% of WNV_NY99_-infected MoDCs and 84.5% of WNV_NSW2011_-infected MoDCs showed CD80 upregulation, and 93.9% of WNV_NY99_-infected MoDCs, 95.8% of WNV_NSW2011_-infected MoDCs showed CD86 upregulation.

**Figure 1:**
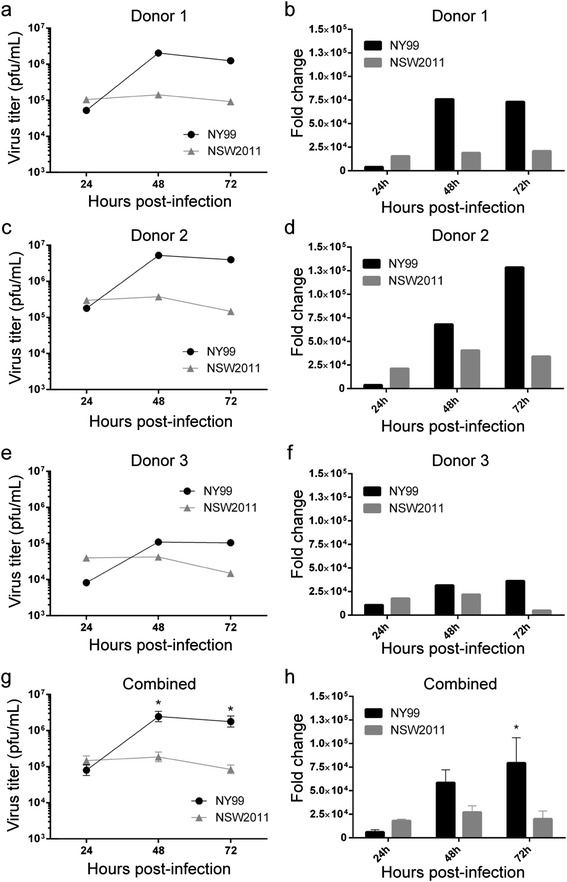
**Replication of WNV**_**NY99**_ **and WNV**_**NSW2011**_ **in human MoDCs.** MoDCs from donors 1 **(a, b)**, 2 **(c, d)** and 3 **(e, f)** were infected with WNV_NY99_ and WNV_NSW2011_ at MOI = 1. Cell culture supernatant was collected at 24, 48 and 72 hours post-infection and virus titer was determined by plaque assay on BHK cells **(a, c, e)**. Total cellular RNA was collected at 24, 48 and 72 hpi and vRNA levels as determined by qRT-PCR were normalised to a combination of three endogenous controls (*TBP, GAPDH and PPIA*), and was expressed as fold change from uninfected samples **(b, d, f)**. The data from all donors (donors 1 to 3) were combined and error bars represent standard error of the mean **(g, h)**. A Student’s t-test was performed to determine statistical significance at each time point (*: P ≤ 0.05).

To compare the ability of WNV_NY99_ and WNV_NSW2011_ to productively infect these initial target cells, MoDCs were infected with WNV_NY99_ and WNV_NSW2011_ (passage 1 stocks grown in Vero76 cells) at a multiplicity of infection (MOI) of 1 and cell culture supernatant and total cellular RNA was harvested at 24, 48 and 72 hpi. Our results revealed that WNV_NY99_ produced similar titers to WNV_NSW2011_ at 24 hpi for donor 1 (Figure 1a) and donor 2 (Figure 1c), but less virus compared to WNV_NSW2011_ for donor 3 (Figure 1e). At later times of infection (48 and 72 hpi), WNV_NY99_ consistently produced higher titers than WNV_NSW2011_ (Figure 1a, c, and e). A similar trend was observed by qRT-PCR analysis of intracellular vRNA levels, showing reduced RNA replication for WNV_NY99_ compared to WNV_NSW2011_ at 24 hpi, but increased RNA replication for WNV_NY99_ compared to WNV_NSW2011_ at 48 and 72 hpi (Figure 1b, d, and f). It was also interesting to note that for all three donors, WNV_NSW2011_ viral titers and vRNA failed to increase after 24 hpi (Figure 1a-f), indicating that WNV_NSW2011_ could not productively replicate in MoDCs while WNV_NY99_ could. In addition, we observed significant variability between human donors, as cells isolated from donor 3 (Figure 1e and f) were more resistant to WNV infection than donors 1 and 2 (Figure 1a-d), which may reflect the high variability in disease symptoms observed in natural human WNV infections [9,15]. When the data from all three donors were combined, WNV_NY99_ produced significantly more virus particles at 48 and 72 hpi (p < 0.05, Figure 1g), and replicated to significantly higher vRNA levels at 72 hpi (p < 0.05, Figure 1h). This confirms the hypothesis that WNV_NY99_ was able to replicate more efficiently than WNV_NSW2011_ in MoDCs, which could possibly translate into increased dissemination in the body, and increased virulence. The enhanced replication of WNV_NY99_ compared to WNV_NSW2011_ was similarly shown in previous studies conducted in our laboratory using commonly used immortalised cell lines [4,16].

**Figure 2:**
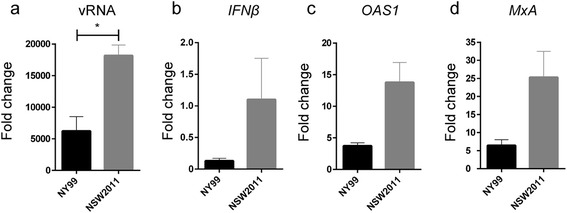
**Early IFN/ISG response to infection with WNV**_**NY99**_ **and WNV**_**NSW2011**_ **in human MoDCs.** MoDCs from three separate donors were infected with WNV_NY99_ or WNV_NSW2011_ at MOI = 1 and total cellular RNA was isolated at 24 hours post-infection. vRNA **(a)**, *IFNβ* **(b)**, *OAS1* **(c)** and *MxA* **(d)** mRNA levels as determined by qRT-PCR were normalised to a combination of three endogenous controls (*TBP, GAPDH and PPIA*), and expressed as fold change from uninfected samples. The results show mean values from three different donors (donors 1 to 3) and error bars represent standard error of the mean. A Student’s t-test was performed to determine statistical significance (*: P ≤ 0.05).

**Figure 3:**
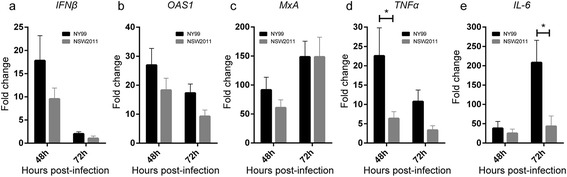
**Late IFN/ISG and pro-inflammatory cytokine response to infection with WNV**_**NY99**_ **and WNV**_**NSW2011**_ **in human MoDCs.** MoDCs from three separate donors were infected with WNV_NY99_ or WNV_NSW2011_ at MOI = 1 and total cellular RNA was isolated at 48 and 72 hours post-infection. *IFNβ* **(a)**, *OAS1* **(b)**, *MxA* **(c)**, *TNFα* **(d)** and *IL-6* **(e)** mRNA levels as determined by qRT-PCR were normalised to a combination of three endogenous controls (*TBP, GAPDH and PPIA*), and expressed as fold change from uninfected samples. The results show mean values from three different donors (donors 1 to 3) and error bars represent standard error of the mean. A Student’s t-test was performed to determine statistical significance at each time point (*: P ≤ 0.05).

It was hypothesized that differences in replication were associated with host innate immune response generated against the viruses. Therefore, qRT-PCR analysis of *IFNβ, OAS1, MxA, TNFα, IL-6* and viral RNA (vRNA) expression was performed in the context of WNV_NY99_ and WNV_NSW2011_ infections. Interestingly, at the early phase of infection (24 hpi), MoDCs infected with WNV_NY99_ had reduced levels of vRNA (p < 0.05, Figure 2a), *IFNβ* mRNA (Figure 2b)***,****OAS1* mRNA (Figure 2c) and *MxA* mRNA (Figure 2d) compared to MoDCs infected with WNV_NSW2011_. In contrast, at the later phase of infection (48 and 72 hpi), RNA levels for WNV_NY99_ were increased (Figure 1h), which likely explains the increased induction of *IFNβ* (Figure 3a)*, OAS1* (Figure 3b) *and MxA* mRNAs (Figure 3c)*,* as well as a significant increase in induction of proinflammatory cytokines *TNFα* (48 hpi p < 0.05, Figure 3d) and *IL-6* (72 hpi p < 0.05, Figure 3e).

The upregulation of *TNFα* and *IL-6* is likely to occur via viral dsRNA activation of TLR3 [9,17,18]. Studies *in vivo* have shown that TLR3 knockout mice were more resistant against WNV encephalitis, and this was linked to decreased TNFα and IL-6 expression [18]. The proposed mechanism of this is that TNFα and IL-6 secreted into the bloodstream from infected leukocytes may induce endocytosis and degradation of tight junction proteins (Claudin-1, JAM-1 and occludin) of the blood brain barrier (BBB), causing breakdown of the BBB and allowing WNV entry into the brain [18-22].

Our finding that WNV_NY99_, but not WNV_NSW2011_, suppresses early viral RNA replication in MoDCs is supported by previous reports. Scherbik *et al.* [23] showed that the pathogenic lineage 1 strain of WNV (Eg101), but not the non-pathogenic lineage 2 strain (W956IC), suppressed early viral RNA replication (but increased viral protein expression), resulting in reduced host innate immune responses at the early phase of infection. This effect of early IFN suppression was also shown for pathogenic vs. non-pathogenic Tick Borne Encephalitis virus (TBEV) strains [24]. These two studies suggest that pathogenic viral strains more effectively suppress early viral RNA replication but increase translation, which has three outcomes that favour virulence; i) decreasing the amount of viral dsRNA to decrease IFN induction, ii) increased viral non-structural protein production to block the JAK/STAT signalling cascade, and iii) more effective viral protein-dependent remodelling of cellular membranes which house replication complexes [23,25].

Taken together, our results have identified that a possible factor leading to higher human virulence of WNV_NY99_ is likely to be decreased viral RNA replication and lower induction of *IFNβ, OAS1 and MxA* early in MoDCs infection. This likely facilitates the enhanced replication of WNV_NY99_ vRNA and dissemination of particles later in infection.
